# Quality of Sleep and Its Correlates among Yemeni Medical Students: A Cross-Sectional Study

**DOI:** 10.1155/2021/8887870

**Published:** 2021-01-18

**Authors:** Bothaina Ahmed Attal, Mohammed Bezdan, Abdulwahab Abdulqader

**Affiliations:** ^1^Faculty of Medicine and Health Sciences, Sana'a University, Sana'a, Yemen; ^2^Centre for Business Research, Cambridge Judge Business School, University of Cambridge, UK; ^3^The Gender-Development Research & Studies Centre (GDRSC), Sana'a University, Yemen

## Abstract

**Background:**

Sleep disturbance is particularly common among medical students worldwide and affects their wellbeing and academic performance. However, little is known about this issue in Yemen. This study looks at sleep quality and its association with personal and life-style factors and self-reported academic performance among medical students at the largest Yemeni university.

**Methods:**

A cross-sectional survey was conducted at Sana'a University, Yemen, in 2017. The Pittsburgh Sleep Quality Index (PSQI), consisting of 19 items and 7 components (score range = 0‐3), was used to assess sleep quality. The summation of the components' scores yields the global PSQI score (range = 0‐21). A global PSQI value higher than 5 indicates poor quality of sleep. Logistic regression was applied to look at relationships.

**Results:**

240 male (41%) and female (59%) medical students took part in the study with 54% being preclinical and 46% clinical with an average age of 23.3 years (SD = 1.7). The mean global score (SD) was 6.85 (2.8), and 68% of the students (*N* = 163) were identified as poor sleepers. The mean global PSQI score (SD) and proportion of poor sleepers were higher among males (7.7 (2.8) and 81%, respectively) than females (6.27 (2.42) and 59.2%, respectively), *p* ≤ 0.001. Good sleep quality was more likely (OR (95% CI)) among females (3.4 (1.3-8.8)), the unmarried (2.8 (1-7.8)), those in good health (2.3 (1.1-4.5)), and nonkhat chewers (4.9 (1.4-17.1)). Nonsmokers were less likely to have good quality sleep compared to occasional smokers (0.185 (0.071-.485)). Stress (30%) and academic workload (21%) were the most commonly reported causes of poor sleep quality. Almost two-thirds of the students (65%) mentioned that disturbed sleep undermined their academic performance.

**Conclusions:**

Poor sleep quality is common among Sana'a medical students and impacts their academic performance. Specific stress management and sleep hygiene promoting programs should be incorporated early on in medical education.

## 1. Introduction

Sleep disturbances incorporate a set of disorders related to initiating and maintaining sleep, excessive somnolence and those related to the sleep-wake schedule, and parasomnias. The latter are dysfunctions associated with sleep, sleep stages, or partial arousals [1]. Sleep disorders in general are increasingly reported in high [2] and low [3] resource settings alike. Poor quality and reduced duration of sleep undermine physical [2, 3] and mental [4, 5] wellbeing as well as the daily functionality and productivity [6]. Disturbed sleep poses considerable health [7] and economic [8] burdens on the communities. Medical students represent one subpopulation that seems to be particularly affected by this problem. A global review revealed that sleep disturbances affect an important proportion of medical students ranging from 41% of the participating students in Iran, 70% in Hong Kong, to 90% in China [9]. Studying medicine entails enduring a large academic workload and involves extended hours of studying and clinical shifts. In addition to the academic demands, other factors can be associated with poor sleep quality among medical students such as late-night internet usage and poor knowledge of sleep hygiene [9, 10]. Inadequate sleep duration and poor quality of sleep among medical students and health professionals in general are associated with poor concentration [11], reduced motor brain functioning [12], impaired behaviour [13], and lower academic performance. Additionally, professionals and medical students who suffer poor sleep tend to have a higher risk of misjudgements [14], accidents, burnout [15], anxiety, depression [16, 17], and substance abuse [9].

In Yemen, information is scarce about the quality and patterns of sleep among medical students and other young populations. Only one published study exists, and this showed that medical students suffered excessive daily sleepiness [18]. This study is aimed at assessing the quality of sleep among medical students in Sana'a University in Yemen and its relationship with personal and life-style characteristics and self-reported academic performance. Information on the size and factors associated with poor sleep quality will inform the public and support students towards adopting healthy sleep practices.

## 2. Materials and Methods

### 2.1. Study Design and Setting

A cross-sectional study was conducted in the Faculty of Medicine and Health Sciences (FMHS) at the public medical school in Sana'a University in October 2017. FMHS is the largest and main medical school in Yemen and receives students from different regions in the country. It offers a 6-year-long Bachelor of Medicine and Bachelor of Surgery degree program equally divided into preclinical and clinical stages. The teaching is conventional in style and based on teacher-centred didactic methods, long lectures, and practical tasks on multiple subjects. Students are frequently assessed with written, oral, and/or practical exams throughout the academic year. Studies have been frequently interrupted during the past 5 years, as a result of war, causing delays in the teaching and exam schedules. The war that started in the country in March 2015 has strained the economy, and security concerns have led to a severe reduction in the university budget and in turn the salaries of staff which has resulted in the departure of many lecturers from the University.

### 2.2. Study Population, Sample Size, and Sampling Technique

A sample size of 239 participants was calculated using the CDC Epi Info™ 3 program, based on a population of 630 medical students, 50% prevalence of poor sleep quality, and 95% level of confidence. A convenience sampling method was used in which students were approached in their lecture hall at the end of the day and were asked to fill out a specifically designed questionnaire. Out of the 265 distributed questionnaires, 240 were found completed on analysis (90.5% response rate). Lecture halls were sampled so that representation would include both male and female students from the third and sixth medical years. These years are the final of the preclinical and clinical years, respectively, and are among the most intense in the medical training program; the third year is marked by a comprehensive exam across basic medical sciences, while the sixth year is an internship year that is concluded by the final MBBS exam.

### 2.3. Data Collection, Tool, and Study Variables

All the data for the sample were collected over a weeklong period. A self-administered questionnaire was distributed to all the participants by a team of trained medical students. The tool was developed in Arabic, the students' native language, and the questionnaire was pretested prior to data collection. It included sections on students' personal and life-style characteristics, the Pittsburgh Sleep Quality Index (PSQI), and, finally, academic performance. The students' characteristics asked covered age, sex, level of study, marital status (single, married, or other), type of residence (with family, on-campus, privately renting, or other), and working besides studying (regular, irregular, or none). Students were also asked to rate their financial status (good, fair, or poor), general health (good, fair, or poor), and dietary intake (regular or irregular). Special habits included smoking (regular, occasional, or none) and khat consumption (daily, occasional, or none). Khat or Catha edulis Forsk is an evergreen plant that contains an amphetamine-like stimulant cathinone. It is been traditionally consumed by chewing the fresh leaves and is mainly used in Yemen and East Africa [19].

The Pittsburgh Sleep Quality Index (PSQI) is a self-rated tool that quantifies sleep quality and identifies disturbances over a month period prior to the survey. Buysse et al. [20] described nineteen individual items, grouped into the following seven components: subjective sleep quality (personal rating of sleep quality), sleep latency, period to fall asleep (in minutes), sleep duration (time of sleep at night in hours), habitual sleep efficiency, sleep disturbances (inability to fall asleep within 30 minutes, waking up in the middle of the night or early morning waking, getting up to use the bathroom, breathing difficulties, cough or loud snoring, feeling too hot or too cold, having bad dreams, having pain, and other), use of sleeping medication, and daytime dysfunction. In addition, information was sought from roommates, if any, to provide collateral reports about the participant's sleep troubles such as loud snoring, long pause between breaths while asleep, or twitching or jerking while asleep. The sleep components each have a score from 0 to 3, with 3 indicating the greatest degree of dysfunction. The cumulative score for each component produces the global PSQI score, which ranges from 0 to 21. A global score of 5 or more indicates poor quality sleep. The original PSQI has been extensively used and evaluated in various settings and has been found to have acceptable psychometric properties [21]. Two Arabic versions were previously developed and tested in separate settings. The PSQI reliability was borderline when measured among a small healthy population (Cronbach′s alpha = 0.65) and acceptable in a clinical sample (Cronbach′s alpha = 0.7) [22]. Both studies reported that the Arabic PSQI had acceptable validity in comparison to other gold standard tools. For the current study, the PSQI was forward and backward translated from the English scale and Arabic language scale to ensure the best possible translation. The Arabic version was pretested prior to the study and modified to ensure its clarity and appropriateness to the Yemeni context. To ensure face validity, the scale was reviewed by a psychologist at the Department of Psychology in Sana'a University. The reliability of the Arabic scale was deemed adequate with Cronbach's alpha of 0.704; please see supplemental file 1 for the component-to-component correlations of the current Arabic version of the PSQI.

Within the data collected, students' perceptions of the reasons for their sleep problems were sought. Students were asked about the extent to which they think that their quality of sleep affects their academic performance in terms of their grades (no, to some extent, or to a great extent) arriving late at lectures (not during the past month, less than once a week, once or twice a week, or three or more times a week), the possibility of dozing-off while studying (none, slight, moderate, or high), and the chance of dozing-off during lectures (none, slight, moderate, or high).

### 2.4. Data Analysis

The personal characteristics relative to the distribution of sleep quality scores were analysed with attention to patterns across demographic and behavioural groups using descriptive statistics. Chi-square tests and Student's *t*-tests were applied to determine if there was a statistically significant difference between categorical and continuous variables, respectively. A binomial logistic regression was applied to estimate the probability of the presence of good quality sleep given the value of the independent variables (personal and life-style factors). A saturated model method was followed using odds ratios (OR) and 95% confidence intervals for all the personal and life-style factors. To better illustrate their association with quality of sleep, the independent variables, apart from the residence, were recoded into dichotomous answers; for example, khat consumption was recoded to chewer/nonchewer and the marital status was recoded as single/ever married. Variables' coding is included in Results. Analyses were performed using IBM's SPSS Statistical Software for Windows (IBM SPSS Version 21, Chicago, IL, USA). All reported *p* values are two-sided and deemed statistically significant at 0.05 or lower.

### 2.5. Ethical Consideration

Ethical clearance was obtained from the Community Medicine Department at the Faculty of Medicine and Health Sciences, Sana'a University. Informed consent was obtained from the students verbally before data collection. No personal identifiers were included in the questionnaire. Anonymity and confidentiality were maintained throughout data collection, storage, and analysis.

## 3. Results

### 3.1. Respondents' Demographics and Life-Style Characteristics

Students were on average 23.3 years old (SD = 1.7), with a slightly higher proportion of females (59%) and third-year students (54%). The majority of the participants were single (83%) and lived with their families (72.5%), which is customary in this age group in Yemen. Over half of the students ranked their financial status as middle (57.7%) and 37% as well-off, and 37% worked during the academic year. In terms of the nutrition and special habits, 42% had their meals irregularly, 14% smoked whether cigarettes or shisha, 37% chewed khat, and 64.5% practised sport whether regularly or otherwise. The vast majority of the students reported that they were in good health (97.5%), and none of them reported mental health issues or current use of psychological medication.  Table 1 shows the personal characteristics of the whole sample and per quality of the sleep group.

### 3.2. Quality of Sleep and Sleep Patterns

Overall, the participants had a mean PSQI global score (SD) of 6.85 (2.8), and 68% (*N* = 163) scored ≥5 indicating poor quality of sleep. The mean PSQI score (SD) varied significantly between the students of poor (8.2 (2.4)) and good (4.1 (.997)) quality of sleep (*t* = 208.377, *p* ≤ 0.001).

**Figure 1:**
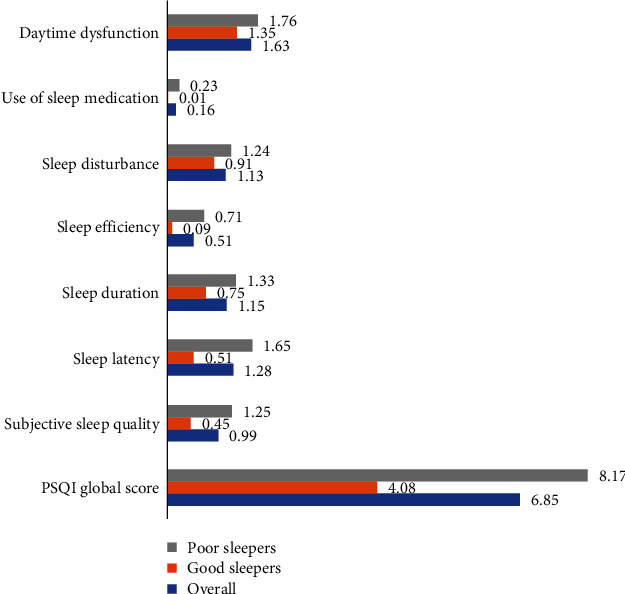
PSQI global and component scores for all the participants and among good and poor sleepers.

The global PSQI and components' scores for the overall sample as well as per quality of the sleep group are shown in Figure 1. As mentioned in Materials and Methods, the higher the scores, the worse the quality of sleep. Figure 1 shows a higher mean score (SD) in the daily dysfunction component of 1.63 (0.75), followed by sleep latency of 1.28 (0.95), while the component score was less with regard to the use of sleep medication of 0.16 (0.55). A similar pattern was seen among both the good and poor sleep quality groups. However, those students who reported poor sleep had higher scores across the PSQI components compared to the good sleepers (*p* ≤ 0.05). For the detailed mean component PSQI scores (SD), please refer to Supplemental File 2.

The quality of sleep measured by the PSQI correlated significantly with the students' subjective reports (*F* = 56.65, *p* ≤ 0.001). The mean PSQI increases significantly across the students' subjective reports of poor quality of sleep. For example, students who thought that their quality of sleep is very good had a mean PSQI score (SD) of 4.7 (2.2) compared to 11.2 (3.3) among those who reported to sleeping very poorly.

### 3.3. Personal and Life-Style Characteristic Predictors of Good Quality of Sleep

Table 2 shows the regression model for factors predictive of good quality sleep. The model is significant (*χ*^2^ (11, 239) = 46.586, *p* < 0.001) with a Nagelkerke *R*^2^ of .248. Sex, marital status, students' health status, chewing khat, and smoking are significant predictors explaining 25% of good sleep quality (*p* < 0.05). Female and single students are three times more likely to be good sleepers than their male (OR = 3.4) and married peers (OR = 2.8). On the other hand, students who do not chew khat are 5 times more likely to have good sleep quality than those that chew khat. In contrast, smoking was inversely correlated with quality of sleep; students who smoke are 5 times more likely to be good sleepers compared to the nonsmokers (inverse OR : 1/0.190 = 5.3). Further examination of the data showed that most of this group is made up of occasional shisha (bubbly) smokers. The univariate association between the quality of sleep and the students' characteristics can be found in Table 1.

### 3.4. Sleep Quality and Patterns among Male and Female Students

Sleep quality varied significantly between males and females in terms of the overall index and for most of the components (see Table 3). Male students had a higher mean global PSQI (SD) (7.7 (2.8)) and a larger proportion of poor sleepers (80.6%) compared to their female peers (6.27 (2.42) and 59.2%, respectively, *p* ≤ 0.001). Also, males tended to sleep at midnight or after (86%) more commonly than females (36%). In addition, males reported shorter duration of sleep; those who slept 6 hours or less constituted 28.6% of the males and 16% of the females, *p* ≤ 0.001. In terms of sleep latency, 59.6% of the students could fall asleep within 30 minutes of going to bed. The proportion of those who took longer than half an hour or more than an hour is larger among males (35.7% and 15.3%, respectively) compared to females (24.6% and 7%, respectively). Three quarters of the students had sleep efficiency of 85% or higher, but overall, a larger proportion of the female students had better sleep efficiency than that of the males (*p* = 0.041). Sleep dysfunction was reported by 92.5% of the students with little sex difference.

### 3.5. Student's Perception of the Reasons for Disturbed Sleep

Mental stress was the most commonly cited single reason (30%) followed by the large academic workload (21%) (Table 4). Male and female students mentioned varying reasons for the sleep disturbances they have experienced. Females were more affected by mental stress (F: 36% and M: 24%), poor physical health (F: 7.5% and M: 4%), and overwork at home (F: 8% and M: 3%) while males mentioned chewing khat (F: 1.5% and M: 20%) and other sex/romantic relationships (F: 3% and M: 9%).

### 3.6. Students' Perceptions of the Association between Poor Sleep and Their Academic Performance

Students were asked about the extent to which their quality of sleep affected their academic performance (see Table 5). Around 65% of the students believe that sleep disturbances undermined their academic performance and grades (*p* = 0.025). Among those who were late three or more times a week, 88% were poor sleepers (*p* = 0.049). Also, 88% and 82.5% of the students who may doze off while studying and during lectures were poor sleepers, respectively (*p* = 0.012).

## 4. Discussion

This study assessed the sleep quality of 240 male and female medical students from the clinical and preclinical levels of education in Sana'a University, in Yemen, using the Pittsburgh Quality of Sleep Index (PSQI). To the best of our knowledge, this is the first published study on quality of sleep in Yemen.

Alarmingly, two-thirds of the student population (*N*: 163) were poor sleepers. Overall, students had a mean PSQI global score (SD) of 6.76 (2.2), which is above the PSQI cut-off for poor quality of 5. These findings are corroborated by the other reports that poor quality of sleep is common among medical students [9]. However, the quality of sleep in the current study is worse than the global averages [23] [24]. The most recent meta-analysis of a pooled global sample of medical students reported a 52.7% prevalence of poor quality sleep (95% CI: 45.3%–60.1%) and a mean PSQI score of 6.1 (95% CI: 5.6 to 6.5) [24]. However, findings vary among countries. In Pakistan, 77% of medical students had poor quality of sleep and a mean PSQI score (SD) of 8.1 (3.1) [25], compared to 58% of Iranian medical students [26]. In Egypt, 53% had poor quality of sleep and a mean PSQI score (SD) of 6 (2.7) [27].

**Figure 2:**
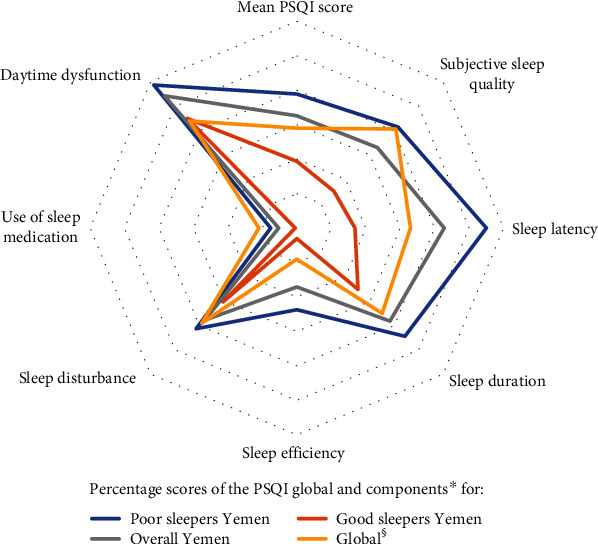
Sleep quality and patterns among medical students in Yemen and at a global level. ^∗^Source data of the PSQI global and components' scores are included in supplemental File 2. ^§^Source: Rao et al. “Sleep Quality in Medical Students: a Comprehensive Meta-Analysis of Observational Studies.” *Sleep Breath*. 2020; 24(3): 1151-1165.

In terms of sleep patterns, Yemeni medical students share a number of similarities with their peers elsewhere.  Figure 2 illustrates the sleep patterns in the current study in comparison with recent global pooled data [24]. For ease of illustration, the mean global and components' scores were converted to percentages (the global PSQI and subcomponent scores were divided by their maximum possible values, i.e., 21 for the global PSQI score and 3 for the component score. The result was multiplied by 100 to produce the percentage, e.g., all sample Yemeni PSQI score (6.85/21)∗100 = 32.9%. The daily dysfunction score (1.63/3)∗100 = 54.3). The higher the value, the worse is the quality of sleep. The overall and component PSQI scores of the current study and of the global pooled sample are included in Supplemental File 2.

Medical students in Yemen and globally are sleep-deprived and suffer daytime dysfunction. However, there is fortunately evidence of a very low use of sleep medicines, probably because of their knowledge on the harmful effects of overuse. It is worth noting that compared to the global pooled sample, Yemeni students sleep for shorter amount of time, take longer to fall asleep, have higher levels of daily dysfunction, have higher levels of sleep disturbances, yet use sleep medicines less frequently and hold higher misperceptions around having good sleep. The latter finding may indicate poor knowledge of sleep hygiene among the Yemeni students and less awareness around their own sleep disturbance.

Personal and life-style characteristics explained 25% of the variation in sleep quality among Yemeni students. The logistic regression model showed that the likelihood of good sleep is threefold higher among female and single students and 5-fold higher among those who do not chew khat. However, quality of sleep is more likely to be better among smokers (*p* < 0.05) (Table 3). The association between gender and sleep quality varied in the literature; reports have found no relationship [28, 29] or inconsistent association [23, 24]. Few other studies found a higher proportion of poor sleepers among females in medical schools [30] and in the general population [31]. But this study adds to the evidence towards male medical students reporting poor quality of sleep more than the females [9]. The main difference in sleep patterns between the male and female Yemeni students was around sleep duration, latency, and efficiency, to the disadvantage of males (see Table 2). Also, these study's findings are consistent with previous reports of a higher prevalence of poor sleep among married students [32, 33], khat chewers [34, 35], and those with physical ill health [31]. However, unexpectedly and unlike previous reports [30, 32], smokers in our study had a 5-fold likelihood of having better quality of sleep than nonsmokers. Further examination of the data showed that students who smoked did so on occasional and infrequent basis, and they smoked shisha rather than cigarettes. Smoking shisha is usually practised in social gatherings. These gatherings, rather than the act of smoking per se, could be a coping mechanism for stress. Unfortunately, pastimes and stress release opportunities are limited in the current conflict situation. Students cited stress and concerns related to study and life as the most common reasons for sleep problems (51%), while physical health problems and internet use formed only 6.5% and 7% of the causes, respectively. The association between stress and psychological disorders on the one hand and sleep disorders on the other has been documented among the medical students [16, 36, 37]. Azad et al. [9] mentioned the study load and early lecture hours may lead to later bedtimes and shorter sleeping hours.

The findings of the current study may be interpreted in the light of a number of limitations. The high prevalence of poor quality sleep in the study may be due to setting the PSQI score of 5 to define poor quality of sleep. Low cut-off values were associated with higher prevalence of poor sleep quality in previous studies [24, 38]. The selection of the third- and sixth-year students preferentially limits the generalizability of the findings to the other years of medical education. In addition, the fact that the third and sixth years are both intense study periods could have masked a possible difference in sleep quality between the pre- and clinical stages of medical studies. Finally, the association between the poor quality of sleep on the one hand and psychological factors and academic performance on the other was based on the students' subjective reports rather than objective measurements.

## 5. Conclusions

This is the first published study in Yemen, and it shows a worryingly high prevalence and severe poor quality of sleep among medical students in Sana'a University, Yemen. Similar to global reports, Yemeni students have a high level of sleep deprivation and daytime dysfunction and a very low use of sleep medicines. Females, those who are unmarried, those who do not chew khat, and shisha smokers are more likely to have a better quality of sleep than their peers. These findings can help inform educators as to the importance of stress management and sleep hygiene promoting programs early on within medical studies. Further research could include an exploration of other populations and could also examine potential psychological factors using standardised tools and would possibly benefit from a qualitative methodology to further explore why the associations found exist.

## Figures and Tables

**Table 1:** Personal and life-style characteristics of the participants overall and per sleep quality group.

Characteristics	Overall *N* = 240	Poor quality *N* = 163	Good quality *N* = 77	*χ* ^2^	*p* value
Age (mean years (SD))	23.3 (1.7)	23.3 (1.6)	23.2 (1.9)	0.249^§^	0.618
Sex				12.252	≤0.01^∗^
Female	142 (59%)	84 (59.2%)	58 (40.8%)		
Male	98 (41%)	79 (80.6%)	19 (19.4%)		
Level of education				0.039	0.89
Third	130 (54%)	89 (68.5%)	41 (31.5%)		
Sixth	110 (45%)	74 (67.3%)	36 (32.7%)		
Marital status				8.043	0.018^∗^
Single	199 (83%)	128 (64.3%)	71 (35.7%)		
Married	32 (13%)	26 (81.3%)	6 (18.8%)		
Other	9 (4%)	9 (100%)	0 (0%)		
Place of residence				1.008	0.799
With the family	174 (72.5%)	115 (66.1%)	59 (33.9%)		
In campus	34 (14%)	25 (73.5%)	9 (26.5%)		
Rented place	28 (12%)	20 (71.4%)	8 (28.6%)		
Other	4 (1.5%)	3 (75%)	1 (25%)		
Health status				4.031	0.133
Good	143 (59.8%)	90 (62.9%)	53 (37.1%)		
Ok	90 (37.7%)	68 (75.6%)	22 (24.4%)		
Poor	6 (2.5%)	4 (66.7%)	2 (33.3%)		
Financial status				6.782	0.034^∗^
High	89 (37%)	52 (58.4%)	37 (41.6%)		
Middle	138 (57.5%)	103 (74.6%)	35 (25.4%)		
Low	13 (5.5%)	8 (61.5%)	5 (38.5%)		
Working while a student				0.503	0.778
No	152 (63%)	103 (67.8%)	49 (32.2%)		
Irregular	67 (28%)	47 (70.1%)	20 (29.9%)		
Regular	21 (9%)	13 (61.9%)	8 (38.1%)		
Chewing khat (*N*: 361)				11.902	0.003^∗^
No	151 (62.9%)	96 (63.6%)	55 (36.4%)		
Occasional	43 (17.9%)	26 (60.5%)	17 (39.5%)		
Regular	46 (19.2%)	41 (89.1%)	5 (10.9%)		
Smoking				2.666	0.264
No	206 (85.8%)	144 (69.9%)	62 (30.1%)		
Occasional	32 (13.3%)	18 (56.3%)	14 (43.8%)		
Regular	2 (0.8%)	1 (50%)	1 (50%)		
Physical exercise (*N*: 360)				1.622	0.444
No	85 (35.4%)	54 (63.5%)	31 (36.5%)		
Irregular	145 (60.4%)	101 (69.7%)	44 (30.3%)		
Regular	10 (4.2%)	8 (80%)	2 (20%)		
Meal intake (*N*: 361)				3.413	0.065
Regular	101 (42%)	62 (61.4%)	39 (38.6%)		
Irregular	139 (58%)	101 (72.7%)	38 (27.3%)		

^§^ *t* value, ^∗^statistically significant, *p* ≤ 0.05.

**Table 2:** Personal and life-style predictors of good quality of sleep among Yemeni medical students.

Variables	OR	95% CI	*p* value
*Age*	1.148	0.806-1.634	0.444
*Sex*			
Female	3.426	1.337-8.779	0.01^∗^
Male	Ref	Ref	Ref
*Educational level*			
Year 3	1.355	0.419-4.384	0.612
Year 6	Ref	Ref	Ref
*Marital status*			
Single	2.846	1.037-7.811	0.042^∗^
Ever married			
*Residence*			0.083
Living with own family	0.299	0.099-0.898	0.031
Campus/private	0.545	0.158-1.883	0.337
Other	Ref	Ref	Ref
*Health status*			
Good	2.268	1.144-4.496	0.019^∗^
Health issues	Ref	Ref	Ref
*Financial status*			
Good	1.924	0.993-3.730	0.053
Poor	Ref	Ref	Ref
*Practising sport*			
Yes	0.575	0.301-1.097	0.093
No	Ref	Ref	Ref
*Chewing khat*			
No	4.893	1.400-17.095	0.013^∗^
Yes	Ref	Ref	Ref
*Smoking*			
No	0.190	0.070-.514	<0.001^∗^
Yes	Ref	Ref	Ref

^∗^Statistically significant, *p* ≤ 0.05.

**Table 3:** Sleep quality and sleep patterns overall and stratified by sex.

Characteristics	Female	Male	All	*χ* ^2^	*p* value
	*N*: 142 (59%)	*N*: 98 (41%)	*N*: 240 (100%)		
*Overall sleep quality*					
Mean PSQI (SD)	6.27 (2.42)	7.70 (3.09)	6.85 (2.80)	16.251^§^	≤0.001^∗^
Percentage poor sleepers	84 (59.2%)	79 (80.6%)	163 (67.9%)	12.252	≤0.001^∗^
*Time of sleep*				71.169^∗^	≤0.001^∗^
Before midnight	91 (64.1%)	14 (14.3%)	105 (43.8%)		
At midnight	37 (26.1%)	34 (34.7%)	71 (29.6%)		
After midnight	14 (9.9%)	50 (51%)	64 (26.7%)		
*Subjective sleep quality*				7.482	0.058
Very good	40 (28.2%)	18 (18.4%)	58 (24.2%)
Fairly good	81 (57.0%)	56 (57.1%)	137 (57.1%)
Fairly bad	18 (12.7%)	16 (16.3%)	34 (14.2%)
Very bad	3 (2.1%)	8 (8.2%)	11 (4.6%)
*Sleep duration (in hours)*				15.593	0.001^∗^
>8	30 (21.1%)	7 (7.1%)	37 (15.4%)
7.1-8	89 (62.7%)	63 (64.3%)	152 (63.3%)
6.1-7	17 (12.0%)	13 (13.3%)	30 (12.5%)
≤6	6 (4.2%)	15 (15.3%)	21 (8.8%)
*Sleep latency (minutes)*				14.31	0.003^∗^
≤15	42 (29.6%)	14 (14.3%)	56 (23.3%)
16-30	55 (38.7%)	32 (32.7%)	87 (36.3%)
31-60	35 (24.6%)	35 (35.7%)	70 (29.2%)
≥60	10 (7.0%)	17 (17.3%)	27 (11.3%)
*Day dysfunction due to sleep*				3.082	0.379
Never	12 (8.5%)	6 (6.1%)	18 (7.5%)		
< once a week	40 (28.2%)	35 (35.7%)	75 (31.3%)		
1-2 times per week	79 (55.6%)	46 (46.9%)	125 (52.1%)		
≥3 times per week	11 (7.7%)	11 (11.2%)	22 (9.2%)		
*Sleep efficiency (%)*				8.251	0.041^∗^
≥85	112 (78.9%)	66 (67.3%)	178 (74.2%)		
75-84	16 (11.3%)	9 (9.2%)	25 (10.4%)		
65-74	5 (3.5%)	8 (8.2%)	13 (5.4%)		
<65	9 (6.3%)	15 (15.3%)	24 (10.0%)		
*Use of sleep medicines*				2.619	0.454
Never	130 (91.5%)	87 (88.8%)	217 (90.4%)
< once a week	6 (4.2%)	7 (7.1%)	13 (5.4%)
1-2 times per week	4 (2.8%)	1 (1.0%)	5 (2.1%)
≥3 times per week	2 (1.4%)	3 (3.1%)	5 (2.1%)

**Table 4:** Students' reasons of sleep disturbances.

Reasons	Overall	Male	Female
	(*N* = 601)	(*N* = 333)	(*N* = 268)
Mental stress	178 (29.6%)	81 (24.3%)	97 (36.2%)
Large academic load	128 (21.3%)	68 (20.4%)	60 (22.4%)
Chewing khat	70 (11.6%)	66 (19.8%)	4 (1.5%)
Personal and family problems	49 (8.2%)	29 (8.7%)	20 (7.5%)
Problems in other sex relationship	37 (6.2%)	29 (8.7%)	8 (3%)
Ill health	32 (5.3%)	12 (3.6%)	20 (7.5%)
Late night internet usage	31 (5.2%)	19 (5.7%)	12 (4.5%)
Fatigue (overwork)	30 (5%)	9 (2.7%)	21 (7.8%)
Accommodation environment	17 (2.8%)	5 (1.5%)	12 (4.5%)
Stimulants (tea, coffee, and energy drinks)	15 (2.5%)	8 (2.4%)	7 (2.6%)
Poor time management	14 (2.3%)	7 (2.1%)	7 (2.6%)

**Table 5:** Students' perception of the extent that poor sleep undermines their academic performance.

	Overall sample^¤^	Poor sleepers^§^	Good sleepers^§^	*χ* ^2^	*p* value
*Arriving late at lectures (N* = 239)				7.839	0.049^∗^
Not during the past month	50 (20.9%)	57 (59.4%)	39 (40.6%)		
Less than once a week	48 (20.1%)	36 (72.0%)	14 (28.0%)		
Once or twice a week	45 (18.8%)	32 (66.7%)	16 (33.3%)		
Three or more times a week	96 (40.2%)	37 (82.2%)	8 (17.8%)		
*Chance of dozing while studying (N* = 240)				1.459	0.692
No chance	29 (12%)	20 (69%)	9 (31%)		
Slight chance	55 (22.9%)	34 (61.8%)	21 (38.2%)		
Moderate chance	75 (31.3%)	51 (68%)	24 (32%)		
High chance	81 (33.8%)	58 (71.6%)	23 (28.4%)		
*Chance of dozing while attending lectures (N* = 239)				10.913	0.012^∗^
No chance	42 (17.6%)	21 (50%)	21 (50%)		
Slight chance	70 (29.3%)	56 (80%)	14 (20%)		
Moderate chance	69 (28.9%)	46 (66.7%)	23 (33.3%)		
High chance	58 (24.3%)	39 (67.2%)	19 (32.8%)		
*Student's sleep quality affects own academic performance (N* = 235)				7.355	0.025^∗^
Yes	64 (27.3%)	46 (71.9%)	18 (28.1%)		
To some extent	91 (38.7%)	68 (74.7%)	23 (25.3%)		
No	80 (34%)	45 (56.3%)	35 (43.8%)		

^¤^Column percentage, ^§^row percentage, ^∗^statistically significant, *p* ≤ 0.05.

## Data Availability

Data are available on reasonable request from the corresponding author.

## Abbreviations

FMHS: Faculty of Medicine and Health Sciences
PSQI: Pittsburgh Sleep Quality Index.

## Supplementary Materials

Supplementary 1 Supplemental file 1: component-to-component correlations of the current Arabic version of the PSQI among medical students, Sana'a University, Yemen.

Supplementary 2 Supplemental file 2: PSQI scores of medical students in Yemen (current study) and in a global pooled sample.
