# Supplementary material for: Quality of Sleep and Its Correlates among Yemeni Medical Students: A Cross-Sectional Study
Source: Sleep Disord. 2021 Jan 18;2021:8887870. doi: 10.1155/2021/8887870 (PMC7841446; doi:10.1155/2021/8887870)
Supplement: Supplementary 1 — Supplemental file 1: component-to-component correlations of the current Arabic version of the PSQI among medical students, Sana'a University, Yemen. [file 8887870.f1.docx]

Supplemental File 1

Component-to-Component Correlation of the Arabic PSQI among medical students, Yemen (N=240)

| **PSQI component** | **C1** | **C2** | **C3** | **C4** | **C5** | **C6** | **C7** | **Global PSQI score** |
| --- | --- | --- | --- | --- | --- | --- | --- | --- |
| **Subjective sleep quality (C1)** |  | .331^**^ | .272^**^ | .249^**^ | .214^**^ | .135^*^ | -0.005 | .610^**^ |
| **Sleep latency (C2)** |  |  | .215^**^ | .253^**^ | .193^**^ | .139^*^ | -0.010 | .637^**^ |
| **Sleep duration (C3)** |  |  |  | .683^**^ | 0.034 | 0.024 | -0.092 | .651^**^ |
| **Sleep efficiency C4)** |  |  |  |  | 0.029 | 0.004 | -0.042 | .689^**^ |
| **Sleep disturbance (C5)** |  |  |  |  |  | .217^**^ | 0.073 | .392^**^ |
| **Use of sleep medication (C6)** |  |  |  |  |  |  | -0.050 | .315^**^ |
| **Daytime dysfunction (C7)** |  |  |  |  |  |  |  | .228^**^ |

**PSQI = Pittsburgh Sleep Quality Index**

***p ≤ .05, ** p ≤ .01**
