# Supplementary material for: Quality of Sleep and Its Correlates among Yemeni Medical Students: A Cross-Sectional Study
Source: Sleep Disord. 2021 Jan 18;2021:8887870. doi: 10.1155/2021/8887870 (PMC7841446; doi:10.1155/2021/8887870)
Supplement: Supplementary 2 — Supplemental file 2: PSQI scores of medical students in Yemen (current study) and in a global pooled sample. [file 8887870.f2.docx]

Supplemental file2

PSQI scores of medical students in Yemen (current study) and in a global pooled sample

|  | Yemen  Mean (SD) | | | Global*  Mean (95% CI) |
| --- | --- | --- | --- | --- |
|  | Poor sleepers | Good sleepers | All Yemen sample | All global pooled sample |
| PSQI global score | 8.17 (2.39) | 4.08 (1.00) | 6.85 (2.80) | 6.1 (5.6-6.5) |
| Subjective sleep quality | 1.25 (0.71) | 0.45 (0.53) | 0.99 (0.75) | 1.22 (1.04–1.41) |
| Sleep latency | 1.65 (0.84) | 0.51 (0.66) | 1.28 (0.95) | 0.99 (0.88–1.11) |
| Sleep duration | 1.33 (0.82) | 0.75 (0.49) | 1.15 (0.78) | 1.05 (0.92–1.18) |
| Sleep efficiency | 0.71 (1.12) | 0.09 (0.33) | 0.51 (0.98) | 0.27 (0.19–0.34) |
| Sleep disturbance | 1.24 (0.52) | 0.91 (0.46) | 1.13 (0.52) | 1.17 (1.01–1.33) |
| Use of sleep medication | 0.23 (0.65) | 0.01 (0.11) | 0.16 (0.55) | 0.33 (0.23–0.43) |
| Daytime dysfunction | 1.76 (0.76) | 1.35 (0.66) | 1.63 (0.75) | 1.32 (1.11–1.53) |

* Source: Rao WW, Li W, Qi H et al. Sleep quality in medical students: a comprehensive meta-analysis of observational studies. Sleep Breath. 2020; 24(3):1151-1165. doi:10.1007/s11325-020-02020-5
